# Stability of Attention Performance of Adults with ADHD over Time: Evidence from Repeated Neuropsychological Assessments in One-Month Intervals

**DOI:** 10.3390/ijerph192215234

**Published:** 2022-11-18

**Authors:** Nana Guo, Janneke Koerts, Lara Tucha, Isabel Fetter, Christina Biela, Miriam König, Magdalena Bossert, Carsten Diener, Steffen Aschenbrenner, Matthias Weisbrod, Oliver Tucha, Anselm B. M. Fuermaier

**Affiliations:** 1 Department of Clinical and Developmental Neuropsychology, Faculty of Behavioral and Social Sciences, University of Groningen, 9712 TS Groningen, The Netherlands; 2 Department of Psychiatry and Psychotherapy, University Medical Center Rostock, 18147 Rostock, Germany; 3 Department of Psychiatry and Psychotherapy, SRH Clinic Karlsbad-Langensteinbach, 76307 Karlsbad-Langensteinbach, Germany; 4 Department of Clinical Psychology and Neuropsychology, SRH Clinic Karlsbad-Langensteinbach, 76307 Karlsbad-Langensteinbach, Germany; 5 Department of Applied Psychology, SRH University Heidelberg, 69123 Heidelberg, Germany; 6 Department of General Psychiatry, Center of Psychosocial Medicine, University of Heidelberg, 69115 Heidelberg, Germany; 7 Department of Psychology, National University of Ireland, Maynooth, W23 F2H6 Maynooth, County Kildare, Ireland

**Keywords:** adult ADHD, selective attention, vigilance, assessment, stability, fluctuation, variability

## Abstract

Neuropsychological assessments of attention are valuable sources of information in the clinical evaluation of adults with attention-deficit/hyperactivity disorder (ADHD). However, it is unclear whether the attention performance of adults with ADHD is stable or fluctuates over time, which is of great importance in the interpretation of clinical assessments. This study aimed to explore the stability of attention performance of adults with ADHD in repeated assessments at one-month intervals. Twenty-one adults diagnosed with ADHD took part in this study by completing selective attention and vigilance tests three times, each one month apart. Test scores of participants were compared with and interpreted based on test norms. A considerable proportion of ‘below average’ performance scores were observed in most of the variables of selective attention and vigilance in all three assessments. Further, selective attention and vigilance performance scores did not differ significantly between the three repeated assessments. Finally, the majority of participants received consistent test score interpretations across the three repeated assessments. This study confirms previous research and highlights abnormal selective attention and vigilance performance in adults with ADHD. Further, this study preliminarily demonstrates relatively stable attention performance across repeated assessments, which has the potential to support clinical assessment, treatment planning, and evaluation.

## 1. Introduction

Attention-deficit/hyperactivity disorder (ADHD) is a childhood-onset neurodevelopmental disorder that lasts into adulthood in the majority of cases and affects about 6% of adults worldwide [1,2]. Deficits in attention are, by definition, a core feature of adult ADHD, and empirical evidence has been presented for deficits in various aspects of attention, including alertness, selective and focused attention, divided attention, sustained attention, and vigilance [3,4,5,6,7,8]. Below-average levels of performance were also observed in various higher-order cognitive functions in adults with ADHD, including response inhibition [9,10], planning [10,11], memory [12,13,14], and decision-making [15,16]. Cognitive dysfunction may interfere with multiple aspects of daily life functioning and may contribute to, for example, poor performance in education and academic settings, poor financial situation, problems at work, traffic accidents and traffic violations, drug abuse, relationship breakup, and problems in socializing [17,18,19,20,21,22].

The close relationship between attention and higher-order cognitive functions has been observed in both clinical samples [23,24,25,26] and non-clinical samples [27,28,29,30] by numerous studies, suggesting that the development of higher-order cognitive functions may be based on the development of basic attention. More recently, the hierarchical relationship between attention and higher-order cognitive functions was reported in patients with ADHD, which underlines the relevance of attention in ADHD [26,31,32]. In this context, research showed that attention might be the foundation of more complex cognitive functions that build upon attention, such as response inhibition, planning, memory, and task-switching. Thus, deficits in attention are significantly associated with and may result in deficits in complex cognitive functions. Further, a network study of cognitive functions on a large sample of adults with ADHD demonstrated that selective attention and vigilance have a central role and high expected influence on other cognitive functions, underscoring the strong interrelation between attention and various other cognitive functions [33].

Considering the fundamental and central role of attention in the clinical neuropsychology of adults with ADHD, an accurate assessment of attention is of great importance for clinical practice, as it may contribute to developing individualized treatment plans and improving the accuracy of treatment evaluation. Neuropsychological performance tests are the mainstay in the assessment of attention functions within the clinical evaluation of adult ADHD. A broad variety of neuropsychological tests are available for neuropsychological practice and research, which have been shown to be sensitive in assessing abnormal attention in both children and adults with ADHD [34,35,36,37]. However, a thorough examination of the evidence provides a more inconsistent picture for application in practice, as most studies derive their conclusion from group comparisons, in which groups of adults with ADHD perform significantly lower than their comparison groups, which does not indicate that all individuals of the respective ADHD group have lower scores than their controls [7,32,38]. Further, while the majority of studies report lower attention scores in adults with ADHD compared to controls in at least some of the attention tests applied, a considerable number of studies also showed intact attention performance in adults with ADHD in other performance measures of attention [39,40,41,42,43]. Finally, the vast majority of attention tests provide several output measures, mostly including indications of both speed and accuracy; however, no consistent picture can be identified whether patients with ADHD typically show deficits in speed, accuracy, or both.

One possible explanation for the consistently inconsistent findings of attention performance in adults with ADHD may be the instability of individuals’ attention performance over time. Intra-individual variability in attention task performance over a period of seconds or milliseconds has been observed repeatedly in individuals with ADHD within the course of a single, one-time assessment [3,44,45,46]. Further, a number of studies demonstrated intra-individual variability over a period of seconds or milliseconds in cognitive functions other than attention in individuals with ADHD, such as working memory [47,48,49] and inhibitory control [49,50,51]. These findings suggest that intra-individual variability may be a ubiquitous and characteristic feature of ADHD and may, at least in part, account for the cognitive heterogeneity observed in adults with ADHD [46,52,53]. Moreover, intra-individual variability in adults with ADHD was not only observed over a period of seconds or milliseconds but was shown more recently also over a period of days and weeks in ADHD symptoms [54,55]. However, compared to time spans of seconds or milliseconds, which have been extensively studied, fewer studies were dedicated to exploring fluctuations in behavior and cognition of adults with ADHD over longer time intervals (e.g., over days, weeks, or months).

Considering that neuropsychological evaluations in clinical practice are usually based on a single, one-time assessment and assume the stability of their findings without repeated assessment, an examination of intra-individual neuropsychological performance fluctuations in adults with ADHD appears relevant and of clinical importance. Thus, the present study aimed to explore the stability of attention performance in adults with ADHD over time through three repeated assessments in one-month intervals. More specifically, a sample of adults diagnosed with ADHD completed a neuropsychological assessment of selective attention and vigilance three times under stable conditions within a time interval of about one month on average. We expected, first, that a substantial number of adults with ADHD perform in the below-average range (T ≤ 36, [56]) of the respective age-representative norm group in the first assessment. Second, we hypothesized that the performances of selective attention and vigilance within each individual were not stable over time but fluctuated from one assessment to the other, which also resulted in different test score interpretations. However, third, we expected that, on a group level, also in the second and third assessment test scores of a considerable proportion of patients with ADHD fall in the below-average range as compared to test norms.

## 2. Materials and Methods

### 2.1. Participants

A total of 21 adults diagnosed with ADHD took part in the present study. All participants were recruited from the Department of Psychiatry and Psychotherapy of the SRH Clinic Karlsbad-Langensteinbach, Germany. Participants were self-referred or referred by local psychiatrists or neurologists to the clinic for a comprehensive ADHD diagnostic because of suspected ADHD in adulthood. The diagnosis of adult ADHD was made jointly by at least two experienced clinicians after a thorough diagnostic assessment. Both clinicians were clinical (neuro)psychologists of the Department of Psychiatry and Psychotherapy of the SRH Clinic Karlsbad-Langensteinbach and mutually agreed upon the diagnostic decision. The diagnostic assessment procedure followed the guidelines for a first-time adult ADHD diagnosis [57]. Specifically, the diagnostic assessment was based on a clinical psychiatric interview enquiring about symptoms and impairments of ADHD and possible comorbidities as outlined in the Diagnostic and Statistical Manual of Mental Disorders (DSM; [1]). The diagnostic assessment further included various types and sources of information, such as reports from schools, and information from partners, parents, and/or employers. An objective indication of impairment was incorporated whenever accessible, e.g., academic failure, unemployment, traffic accidents, drug use, relationship breakups, divorces, etc. To assess the severity of ADHD symptoms, participants completed 2 self-report scales that were developed to quantify retrospective and current ADHD symptoms, i.e., the short version of the Wender Utah Rating Scale (WURS-K; [58]) and the Conners’ Adult ADHD Rating Scales—Self-Report: Long Version (CAARS-S:L; [59]). Among those 21 individuals diagnosed with ADHD, 2 met the diagnostic criteria of the predominantly inattentive presentation of ADHD, 18 met the diagnostic criteria of the combined symptom presentation, while the symptom presentation of one individual was not reported. Moreover, seven of the 21 individuals were additionally diagnosed with one or more comorbid psychiatric disorders, including mood disorders (*n* = 6), addiction disorders (*n* = 1), anxiety disorders (*n* = 1), and post-traumatic stress disorder (*n* = 1). Twelve of the 21 individuals were currently treated with stimulant medication under stable conditions on all three assessment days. The remaining 9 participants did not take stimulant medication on any of the assessment days. Descriptive information and ADHD symptom scores are presented in Table 1.

### 2.2. Materials

This study is part of a larger project which comprises an extensive battery of measures for the assessment of symptoms, impairments, and cognitive functioning of adults with ADHD. In the following sections, only the measures relevant to this study are described.

#### 2.2.1. Self-Report Scales of ADHD Symptoms

The German short version of the WURS-K was administered to assess ADHD symptoms in childhood [58]. This scale consisted of a total of 25 items (21 items assessing symptoms, 4 control items), each scored on a 5-point Likert scale ranging from 0 (not at all or very slightly) to 4 (very much). Participants rated each item based on the recall of their childhood experiences. The sum score of 21 items (except for the 4 control items) was calculated to assess the severity of retrospective ADHD symptoms.

The German version of CAARS-S:L was applied to assess the severity of current ADHD symptoms [59]. The CAARS-S:L includes 66 items, each scored on a 4-point Likert scale ranging from 0 (not at all/never) to 3 (very much/very frequently). A number of subscales can be derived from the CAARS-S:L. For the present study, the sum scores of three subscales that assess ADHD symptoms as listed in the DSM-IV ([60]) were calculated and reported, i.e., the DSM-IV subscales for inattentive symptoms, hyperactive/impulsive symptoms, and total symptoms.

#### 2.2.2. Assessment of Selective Attention and Vigilance

Selective attention and vigilance were assessed with two computerized tests of the Vienna Test System (VTS; [61], including the test perception and attention functions: selective attention (WAFS) and the test perception and attention functions: vigilance (WAFV). These two tests have been shown valid and sensitive in revealing attention deficits in various clinical populations and are commonly applied in the clinical neuropsychological evaluation of adult ADHD and related disorders [62,63,64].

##### Selective Attention

The test WAFS was used to assess selective attention. In this test, three kinds of geometric stimuli (circles, squares, and triangles) that may get lighter or darker or stay the same were presented on the screen. Participants were asked to respond as quickly as possible to changes in circles and squares (a circle gets lighter or darker, a square gets lighter or darker) by pressing the response button. No response is needed when a triangle gets lighter or darker. Each stimulus was presented for 1500 ms, and a change may take place after 500 ms. The interstimulus interval was 1000 ms. A total of 144 stimuli were presented, 30 of which were targets. The test duration is about 8 min. The mean reaction time (RT), the logarithmic standard deviation of the reaction times (SDRT), and the number of missed reactions (omission errors) were recorded. Adult norms are accessible consisting of 295 individuals representing the general population (46.4% men; 53.6% women) aged between 16 and 77 (median = 39; sd = 15.1). All individuals in the normative group performed tests in the German language. The internal consistency (Cronbach’s α) of this test is excellent, with 0.95 in the normative sample.

##### Vigilance

The test WAFV was administered to assess vigilance. In this test, squares that may get darker or stay the same were presented one by one on the screen. Participants were asked to press the response button as quickly as possible whenever a square got darker. The frequency of targets (squares getting darker) is 5%. Each stimulus was presented for 1500 ms, and a change may take place after 500 ms. The interstimulus interval was 500 ms. A total of 900 stimuli were presented, 50 of which were targets. The test duration is 30 min. The mean RT, the logarithmic SDRT, and the number of omission errors were recorded. The WAFV and the WAFS are evaluated based on the same normative group, consisting of 295 individuals representing the general population (46.4% men; 53.6% women) aged between 16 and 77 (median = 39; sd = 15.1). All individuals in the normative group performed tests in the German language. The internal consistency (Cronbach’s α) of this test is excellent, with 0.96 in the normative sample.

### 2.3. Procedure

All participants were invited to take part in the study on a voluntary basis. It was stressed to all participants that the study was for research purposes only and will not affect their clinical evaluation and treatment. All participants signed the written informed consent before taking part in the study. The neuropsychological assessment of selective attention and vigilance was performed three times on three different assessment days. The time interval between the different assessments was, on average, 1 month from each other, ranging from 21 to 49 days, and in 90% of the cases ranging from 21 to 35 days. The assessments per person took place, as much as possible, on the same day of the week and at the same time of the day. All assessments were performed in a quiet environment at the Department of Psychiatry and Psychotherapy of the SRH Clinic Karlsbad-Langensteinbach, Germany. The order of the administration of all tests (including measures of selective attention and vigilance) was randomized across participants but kept constant for each individual across all three assessment days. Self-report symptom scales were completed only once, i.e., prior to the first attention assessment. Participants were rewarded with 60 euros upon completion of all three assessments. Participants who were included in the present study were assessed between January 2019 and June 2021. The study was approved by the ethical review board of the medical faculty of the University of Heidelberg, Germany (protocol code: S-588/2018, date: 1 October 2018).

### 2.4. Statistical Analysis

Missing values occurred in 2.7%, 4.7%, and 13.8% of the data for the first, second, and third assessments, respectively, and the missing values were not replaced. Descriptive statistics of attention performance scores are presented for all participants. Moreover, T-scores based on representative normative data as provided by the test publisher are computed [61]. T-scores equal to or lower than 36 are defined as ‘below average’ performance [56]. The percentages of participants scoring in the below-average range (T ≤ 36) are calculated per test variable for each of the three assessments. Further, attention performance scores are compared between the three assessments using nonparametric statistics (i.e., Friedman test). Post hoc pairwise comparisons are computed using Wilcoxon signed-rank tests. To control the alpha inflation in multiple testing, a Bonferroni adjusted significance level of *p* < 0.017 (*p* = 0.05/3) was applied. The magnitude of pairwise differences is indicated using effect size *Cohen’s r*, with *r* = 0.1 indicating a small effect, *r* = 0.3 indicating a medium effect, and *r* = 0.5 indicating a large effect [65].

Moreover, the absolute values of individual T-score differences between any two of the three assessments are calculated for each test variable. Further, to examine whether test score interpretations differed from one assessment to another, the percentage of consistent test score interpretations is indicated for each comparison. Consistent test score interpretation is considered if both test scores indicate below-average performance (T ≤ 36) or both test scores indicate no below-average performance (T > 36). The percentage of consistent test score interpretation is calculated by the number of participants who received consistent test score interpretation divided by the total number of participants. The percentage of consistent test score interpretations across three assessments is also calculated for each variable. Finally, Spearman correlation coefficients are calculated to indicate the association between test scores at any two of the three assessments and a Bonferroni adjusted significance level of *p* < 0.017 (*p* = 0.05/3) was applied.

## 3. Results

**Figure 1:**
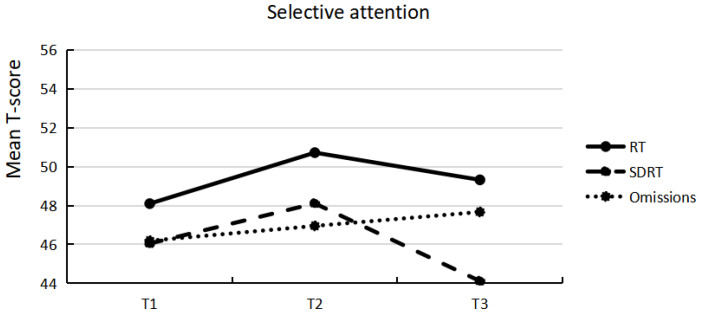
Mean T-score of variables of selective attention at each of the three assessments.

**Figure 2:**
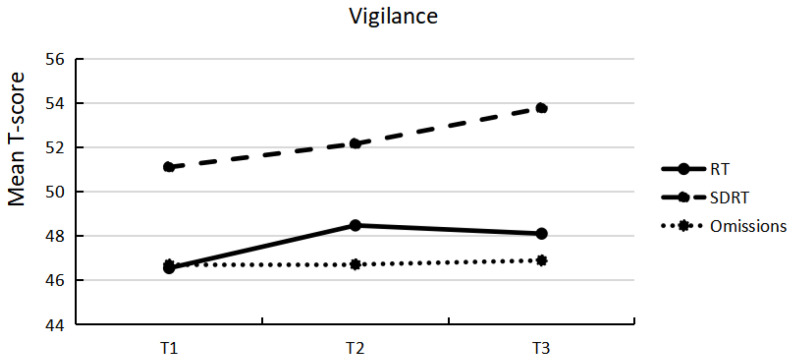
Mean T-score of variables of vigilance at each of the three assessments.

Descriptive statistics of attention performance for the three assessments and the percentages of participants who scored below average (T ≤ 36) are presented in Table 2. A comparison to test norms indicates a higher percentage of participants scoring in the below-average range, in particular in the selective attention task. Figure 1 and Figure 2 illustrate mean T-scores below 50 for most of the variables of the selective attention and vigilance task and mean T-scores slightly above 50 for SDRT of the vigilance task. Figure 1 and Figure 2 visually depict that no meaningful changes occurred from one time point to another on a group level. Furthermore, comparing attention performance over time (T1, T2, and T3) revealed no significant differences. Pairwise comparisons confirmed non-significant differences, with effect sizes ranging from negligible to small size (Table 2).

Further, individual changes are indicated by absolute T-score differences from one time point to another. Descriptive statistics of absolute T-score differences between assessments are presented in Table 3. Results indicate that the mean T-score difference ranges from 3.2 to 8.9, with the smallest difference observed between the second and the third (T2–T3) assessment in the omissions of the selective attention task and the largest difference observed between the second and the third (T2–T3) assessment in the SDRT of the vigilance task. In addition, the majority of participants received the same test score interpretation as below average (T ≤ 36) or no below average (T > 36), with at least 74% of participants being consistent in their test score interpretation across all three assessments (see Table 3). When comparing test score interpretations between any two assessments, the consistency rate is above 80% for the majority of measures and comparisons (Table 3). In total, 20–25% of participants received inconsistent test score interpretations. Finally, correlation analyses revealed that the RTs of both selective attention and vigilance are significantly correlated (large effect size) between any two of the three assessments (*p* < 0.01). However, for SDRT, a significant correlation is observed only between the first and the third assessment of selective attention (*p* < 0.05; large effect). For omission errors, significant correlations are observed between any two of the three assessments of selective attention (medium to large effect size) but only between the first and the second assessment in the vigilance task (medium effect size).

## 4. Discussion

This study aimed to explore the stability of attention performance over time in adults diagnosed with ADHD by repeatedly assessing selective attention and vigilance three times with a one-month time interval between each assessment. Results confirmed previous research showing that a considerable proportion of individuals with ADHD score in the below-average range in both selective attention and vigilance. Furthermore, both performance scores and test score interpretations (below average or not) remained relatively stable over time.

Inspecting the attention performance of adults with ADHD revealed that the percentage of participants who scored in the below-average range was higher than expected from a representative norm group (≥ 8%, [56]) in most of the attention measures assessed, in particular in selective attention. Mean T-scores were below 50 for most variables (except for the SDRT of the vigilance task with mean T-scores slightly higher than 50), although the percentage of participants scoring in the below-average range in the variability of reaction times and omission errors of the vigilance tests was not as high as in the reaction times of the vigilance task. These results, thus, confirm previous findings that selective attention and vigilance deficits may be characteristic of adult ADHD [5,7,66,67] and that neuropsychological performance tests are sensitive instruments to demonstrate attention deficits in the clinical evaluation of adults with ADHD [35,36,37,62].

Different from our expectations, the comparisons of attention performance between the three assessments revealed relatively stable scores with nonsignificant group differences of negligible to small size. Moreover, most individuals deviated only slightly in the performance scores, as shown by individual T-score differences of about 5 points (ranging from 3.2 to 8.9) from one assessment to another, which further supports the notion of stable selective attention and vigilance dysfunction over time. Prior to data analysis, we expected selective attention and vigilance performance to fluctuate from one assessment to another based on research showing cognitive performance fluctuations in tasks over seconds and milliseconds in individuals with ADHD [44,46,47,48,50,51], as well as inconsistent findings on attention performance in individuals with ADHD [5,38,40,41,43]. The relatively stable attention performance over time observed in the present study suggests that inconsistent findings across previous studies in adults with ADHD may more likely be related to external factors, such as differences in test selection, sample composition, or clinical characteristics, rather than reflecting unstable cognitive abilities. Further, preliminary conclusions could be drawn that attention performance may fluctuate over seconds or milliseconds in individuals with ADHD within the course of a single, one-time assessment [3,44,45,46], but overall attention performances may be stable across assessments with longer time intervals. A neural basis for performance fluctuations within tasks in adults with ADHD was suggested in imaging research by showing a link between greater reaction time variability in adults with ADHD and reduced activation in frontoparietal brain regions and anterior cingulate gyrus [53,68,69,70]. However, this activation pattern may be similar when repeatedly performing the same tasks at different time points, resulting in relatively stable performance scores, as observed in this study. Further, this study provides implications regarding the test-retest reliability of the applied instruments on clinical samples of adults with ADHD. Although good reliability estimates of the applied tests were provided by the test publisher, most of the reliability analyses stem from non-clinical samples. Thus, evidence of test-retest reliability of these tests on clinical samples is scarce. Consistent and stable attention performance across three repeated assessments as observed in the present study, thus, support the test-retest reliability of these attention tests in clinical samples. Moreover, correlation analyses demonstrated the largest associations between variables of reaction time, whereas considerably smaller and mostly non-significant associations were found between variables representing SDRT and omissions. Although previous research in this field failed to identify a specific variable type (including RT, SDRT, omissions and commissions) that stands out to be most sensitive in detecting vigilance deficits in adult ADHD [62], current data indicate that measures of reaction time seem to be most stable across time.

More importantly for clinical practice, test score interpretations were consistent across the three assessments for the majority of participants. Specifically, more than 74% of participants received consistent test score interpretations (below or no below-average performance) across all three assessments, and more than 80% of participants received consistent test score interpretations between any two assessments for most variables. Consistent test score interpretations are encouraging for clinicians by providing an evidence base that the results of a one-time neuropsychological attention assessment may be well suited as a basis for clinical evaluation, treatment planning, and treatment evaluation. Of note, there are still about 20-25% of participants who received inconsistent test score interpretations across the different assessments, which may indicate unstable attention performance over time. For these cases, a one-time attention assessment may bias or distort the interpretation of cognitive performance scores, and it is advisable to integrate results from neuropsychological performance assessment with other clinical measures (of attention) in order to receive a comprehensive and valid understanding of the individual’s functioning [71,72,73]. However, also for those participants receiving inconsistent test score interpretations, it must be stressed that T-score differences remained rather small (maximum difference of 8.9 points), and inconsistent test score interpretations mainly resulted from the dichotomous nature of test score interpretations as applied in this study. In addition, another possible explanation for the inconsistent test score interpretations observed in 20–25% of participants could be the occurrence of regression towards the mean [74], which is shown by participants yielding an extremely low or high score in the first assessment, but a score closer to the mean in a reassessment (i.e., extreme scores get less extreme in a reassessment). The occurrence of such effects of regression towards the mean may also lead to inconsistent test score interpretations across three assessments. Finally, the majority of participants (12 of the 21) were currently treated with stimulant medication, so it could be speculated that the stability of attention performance observed in most cases reflects the cognitive ability status of individuals who are already diagnosed and treated for ADHD, but not individuals who are still seeking a clinical diagnostic check-up.

## 5. Limitations and Future Directions

Of note, the data of this study need to be interpreted in the context of several limitations. Most importantly, the sample size of this study is relatively small and may lead to low statistical power. Thus, the conclusions of this study should be taken as preliminary and require replication on larger and more homogenous samples. Larger samples would yield more robust findings and may allow the investigation of moderating factors, such as symptom presentation, comorbidities, or medication status. Support for the robustness of our results is given by the good psychometric properties of our instruments and a similar distribution of scores in one-time assessments on independent and large clinical samples [32,33]. Second, some of the participants (7 of 21) were additionally diagnosed with one or more comorbid psychiatric disorders other than ADHD, which may confound our findings. However, it must be noted that our sample represents a naturalistic sample of patients with ADHD and how it commonly occurs in clinical settings, in which comorbid psychiatric disorders are the rule rather than the exception. Thus, controlling or removing comorbidity would result in unrealistic samples. Additionally, as symptoms of inattention and hyperactivity also occur in many other disorders than ADHD, the findings of this study may also hold for other related conditions with overlapping symptoms, which need to be addressed in future research. Third, administrative difficulties in implementing the repeated assessment design in clinical practice resulted in non-identical time intervals between assessments. Even though differences were kept as minimal as possible, it must be noted that variation in time intervals may confound results on performance stability. Forth, implications on test-retest reliability of tests used in this study in clinical samples should also be taken into consideration while interpreting results. Future studies on independent and larger clinical samples could benefit us in getting a more accurate understanding of test-retest reliability in clinical populations. Fifth, the findings of this study are restricted in a way that they are based on selective attention and vigilance performance as assessed by the Vienna Test System. Future replication studies would benefit from including also other measures to gain information on the specificity/generalization of the findings. Finally, test score interpretations contrasted ‘below average’ performance with all other performance scores, which neglects more nuanced and fine-grained performance evaluations that are possible on larger samples, e.g., also considering cognitive strengths in above-average scores.

## 6. Conclusions

This study confirms previous research that a considerable proportion of adults with ADHD show below-average selective attention and vigilance performance. Moreover, this study highlights that selective attention and vigilance performance did not differ significantly between three repeated assessments in one-month intervals. In this context, the majority of individuals showed only minor T-score differences between the three assessments and received consistent test score interpretations. Pending future replication on larger samples, this study forms an empirical evidence base relevant for clinical practice as it suggests that the results of a one-time attention assessment may be sensitive in revealing attention deficits and may be stable over time for the majority of individuals, thus being useful to guide individual treatment planning and evaluation.

## Figures and Tables

**Table 1:** Descriptive information and ADHD symptoms of all participants.

	Adults with ADHD (*n* = 21)				
Sex (Male/Female)	8/13				
Education (1/2/3/4/5/6) ^a^	0/1/8/6/5/1				
	Min	Max	Median	Mean	SD
Age (in years) ^b^	20	65	47.5	46.1	11.6
Childhood ADHD symptoms ^c^	11	57	41	39.3	13.1
Current ADHD symptoms ^d^					
Inattention	1	22	13	13.2	6.2
Hyperactivity/Impulsivity	3	24	13	12.2	6.0
Total symptoms	6	46	25	25.5	10.7

Note: ^a^ Education (1/2/3/4/5/6) = Basic schooling with no formal degree (less than 9 years of schooling)/ Secondary school (usually 9–10 years of schooling)/ Secondary school with additional vocational training (usually 10–12 years of schooling)/ Secondary school with university entrance qualification (usually 12–13 years of schooling)/ University degree (usually 16–17 years of schooling)/Not reported. ^b^ Data was missing for one participant. ^c^ Measured with the short version of the Wender Utah Rating scale (WURS-K); data were missing for eight participants. ^d^ Measured with the Conners’ Adult ADHD Rating Scales—Self-Report: Long Version (CAARS-S:L); data were missing for eight participants.

**Table 2:** Attention performance of individuals with ADHD (*n* = 21) across the three assessments T1, T2, and T3.

Attention Performance Test Scores	T1			T2			T3			Friedman Test		Pairwise Comparisons ^a^		
												T1 vs. T2	T1 vs. T3	T2 vs. T3
	Median	IQR	% Below Average ^b^	Median	IQR	% Below Average ^b^	Median	IQR	% Below Average ^b^	*X* ^2^	*p*	*Cohen’s r* ^c^	*Cohen’s r* ^c^	*Cohen’s r* ^c^
Selective attention ^d^—RT	394.00	158.00	23.8	354.00	136.00	14.29	339.00	166.50	21.05	0.12	0.94	0.15	0.02	0.12
Selective attention ^d^—SDRT	1.24	0.12	19.05	1.20	0.07	10.53	1.25	0.10	17.65	0.92	0.63	0.09	0.09	0.25
Selective attention ^d^—Omissions	0	1.00	19.05	0	0.75	15.00	0	0.25	16.67	0.26	0.88	0.08	0.09	0.11
Selective attention ^e^			33.33			19.04			36.84					
Vigilance ^f^—RT	440.00	109.50	15.00	421.00	150.00	23.81	434.00	163.00	21.05	3.35	0.19	0.13	0.09	0.05
Vigilance ^f^—SDRT	1.24	0.08	5.26	1.24	0.09	5.26	1.21	0.06	0	0.52	0.77	0.13	0.25	0.08
Vigilance ^f^—Omissions	0	1.00	15.00	1.00	1.00	9.52	1.00	1.00	0	0.35	0.84	0.09	0.06	0.05
Vigilance ^e^			20.00			38.10			21.05					

Note: T1 = First assessment, T2 = Second assessment, T3 = Third assessment. IQR = Interquartile range. ^a^ Post hoc tests were performed using Wilcoxon signed-rank tests. None of the comparisons indicated significance on a Bonferroni-adjusted significance level of *p* = 0.017. ^b^ Percentage of participants who scored in the below-average range (T score ≤ 36). ^c^ Based on *Cohen’s* criteria for *r*: *r* = 0.1 indicates a small effect, *r* = 0.3 indicates a medium effect, and *r* = 0.5 indicates a large effect. ^d^ Selective attention was assessed using the Perceptual and Attention Functions—Selective attention (WAFS). ^e^ Below average performance in selective attention/vigilance is defined by below average performance in at least one variable of its test variables. ^f^ Vigilance was assessed using the Perceptual and Attention Functions—Vigilance (WAFV).

**Table 3:** Stability of attention performance and correlations between attention scores of the three assessments of individuals with ADHD (*n* = 21).

Attention Performance Test Scores	T1–T2 ^a^				T1–T3 ^a^				T2–T3 ^a^				% Consistent Test Score Interpretations across the Three Assessments
	Range	M	% Consistent Test Score Interpretations ^b^	*r* ^c^	Range	M	% Consistent Test Score Interpretations ^b^	*r* ^c^	Range	M	% Consistent Test Score Interpretations ^b^	*r* ^c^	
Selective attention ^d^—RT	2–17	5.86	81	0.828 *	0–19	4.95	79	0.833 *	0–9	4.58	95	0.927 *	79
Selective attention ^d^—SDRT	1–23	8.79	89	0.369	1–22	7.82	76	0.556	0–21	8.00	88	0.265	75
Selective attention ^d^—Omissions	0–23	4.10	80	0.569 *	0–23	4.78	83	0.489	0–14	3.24	100	0.592 *	82
Vigilance ^e^—RT	0–17	4.35	80	0.779 *	0–29	6.05	84	0.591 *	0–13	5.53	84	0.784 *	74
Vigilance ^e^—SDRT	1–24	8.59	88	0.379	2–19	6.65	95	0.441	1–33	8.88	94	0.238	88
Vigilance ^e^—Omissions	0–23	5.85	75	0.465	0–23	5.95	85	0.221	0–23	5.05	89	0.290	74

Note: T1 = First assessment; T2 = Second assessment; T3 = Third assessment. ^a^ T1–T2 = Mean of absolute values of T-score differences between the first and the second assessment; T1–T3 = Mean of absolute values of T-score differences between the first and the third assessment; T2–T3 = Mean of absolute values of T-score differences between the second and the third assessment. ^b^ Percentage of participants who received consistent test score interpretations across two assessments. ^c^ *r* indicates the Spearman correlation between the two assessments. ^d^ Selective attention was assessed using the Perceptual and Attention Functions—Selective attention (WAFS). ^e^ Vigilance was assessed using the Perceptual and Attention Functions—Vigilance (WAFV). * Correlation is significant at the Bonferroni adjusted level, *p* < 0.017.

## Data Availability

The data that support the findings of this study are available from the corresponding author upon reasonable request.
